# Endothelial Alterations in Systemic Lupus Erythematosus and Rheumatoid Arthritis: Potential Effect of Monocyte Interaction

**DOI:** 10.1155/2017/9680729

**Published:** 2017-05-04

**Authors:** Laura Atehortúa, Mauricio Rojas, Gloria M. Vásquez, Diana Castaño

**Affiliations:** ^1^Grupo de Inmunología Celular e Inmunogenética, Instituto de Investigaciones Médicas, Facultad de Medicina, Universidad de Antioquia (UdeA), Calle 70 No. 52-21, Medellín, Colombia; ^2^Unidad de Citometría, Facultad de Medicina, Sede de Investigación Universitaria, Universidad de Antioquia (UdeA), Calle 70 No. 52-21, Medellín, Colombia

## Abstract

Patients with systemic autoimmune diseases such as rheumatoid arthritis (RA) and systemic lupus erythematosus (SLE) are prone to develop atherosclerosis and cardiovascular diseases five times more often than the general population; this increase in frequency could be partially explained by an increase in the macrovasculature endothelial damage. In these autoimmune diseases, a microvascular endothelial injury has also been reported in different organs and tissues, especially in sites where ultrafiltration processes occur. Different components that are characteristic to the immunopathology of RA and SLE could be involved in the endothelial cell activation, permeability increase, functional alteration, and vascular injury. Circulating immune complexes (IC) detected in SLE and RA have been proposed to participate in the endothelial injury. In the vascular environment, IC can generate different responses that could be mediated by monocytes, because these cells have patrolling and monitoring functions on the endothelium. However, with certain stimuli such as TLR ligands, the monocytes are retained in the lumen, releasing proinflammatory mediators that participate in the endothelial damage. This paper aims to review some aspects about the endothelial activation and dysfunction in the context of SLE and RA, as well as the potential role that monocytes apparently play in this process.

## 1. Introduction

Endothelial cells had not been previously considered as a key component of the immune system; however, there are growing evidences that show the involvement and modulation of these cells through innate and adaptive immune responses [1, 2]. This initial question about their essential immunological contribution was mainly due to the endothelial diversity and the variety of functions performed by these cells in the cardiovascular system, for example, in regulating homeostasis and blood flow [1–4]. Endothelial cell activation and perturbation have been associated with different immunopathological processes, such as atherosclerosis, diabetes, pulmonary hypertension, rheumatoid arthritis (RA), systemic lupus erythematosus (SLE), hemoglobinopathies, and certain infectious diseases like dengue, among others [1, 3, 5, 6].

In the chronic and systemic inflammatory processes presented in SLE and RA, a gradual deterioration of different organs, mainly the endothelial injury leads to an increased risk of developing complications such as atherosclerosis and cardiovascular diseases, which are the most common causes of premature mortality in patients with SLE and RA [5]. Different genetic, epigenetic, environmental, hormonal, and immunological factors have been involved in the establishment of these diseases [7]. In both cases (SLE and RA), there is immune complex (IC) formation and deposit in circulation, which could cause direct or indirect endothelial damage. IC are generated because the autoantibodies recognizes all potential autoantigens present in the blood, in soluble form or as part of the vesicular structures such as apoptotic cells (AC) and microparticles (MP) [8–10].

It has been established that monocytes and macrophages play a central role in the immunopathology of SLE and RA, principally by removing autoantigens and IC, migrating into the site of inflammation, and by the production of proinflammatory factors such as interleukin- (IL-) 1*β* and tumor necrosis factor- (TNF-) *α* and chemokines like IL-8, reactive oxygen species (ROS), and nitric oxide (NO) [11, 12]. Monocytes have been described as the main patrolling cells of the endothelium maintaining the integrity of this tissue in basal conditions; however, the IC presence and deposit in the vasculature might modulate the endothelial microenvironment, changing the interaction and responses of monocytes to this tissue and compromising the endothelium integrity. Therefore, monocytes should participate in endothelial dysfunction presented in RA and SLE patients. In this paper, we reviewed the information regarding the activation and endothelial alterations in the context of SLE and RA, and the current knowledge of how monocytes participate in both the activation of endothelial cells, as well as their damage. In addition, we propose a possible mechanism by which the monocyte subpopulations interact with the endothelial cells favoring their alterations in the macro- and microvascular context of SLE and RA; however, this model is limited due to the scarce information available regarding this topic at this moment.

## 2. Overview of Endothelial Cells

Endothelial cells coat the inner wall of the blood vessels forming a single layer of cells called endothelium. These cells can be part of the macrovasculature (large vessels with an internal diameter ≥ 100 *μ*m), which is made up of three layers: intima, media, and adventitia [13]. The endothelial cells are also part of the microvasculature (small vessels with an internal < 100 *μ*m) that includes arterioles, venules, and capillaries, integrated with endothelial cells and pericytes (perivascular contractile cells “*Rouget cells*”) to maintain the integrity of this vascular wall [13].

From the earliest stages of embryonic organ development, it is apparent that vessels are not merely conduits for blood, nutrients, gas exchange, and waste disposal; instead, they are essential components of different tissues for their functions and specialization. Therefore, endothelial cells specialize in each tissue, displaying unique organ-associated antigens. For this reason, they are very heterogeneous and have specialized roles in different locations as well as show variations in response to stimuli, injury, and repair which determine the disease patterns [4]. Nowadays, growing evidence in this field shows that the microvascular endothelial cells (MIEC) differ in phenotype, gene expression, and physiology than macrovascular endothelial cells [14]. Human umbilical vein endothelial cells (HUVEC) proliferate well in serum-containing medium and seem to be less demanding in endothelial growth factors than MIEC, reflecting differences in the machinery regulating cell cycle between these cells [15]. The amount of vasoactive substances (endothelin-1, thromboxane, angiotensin II, and prostacyclin) released into the culture medium by these cells is also different, for example, MIEC secrete 2-fold higher quantity of angiotensin-II than HUVEC [16]. Therefore, endothelial cells are morphologically and functionally heterogeneous with the greatest differences between those that are from the macro- and microcirculation.

**Figure 1:**
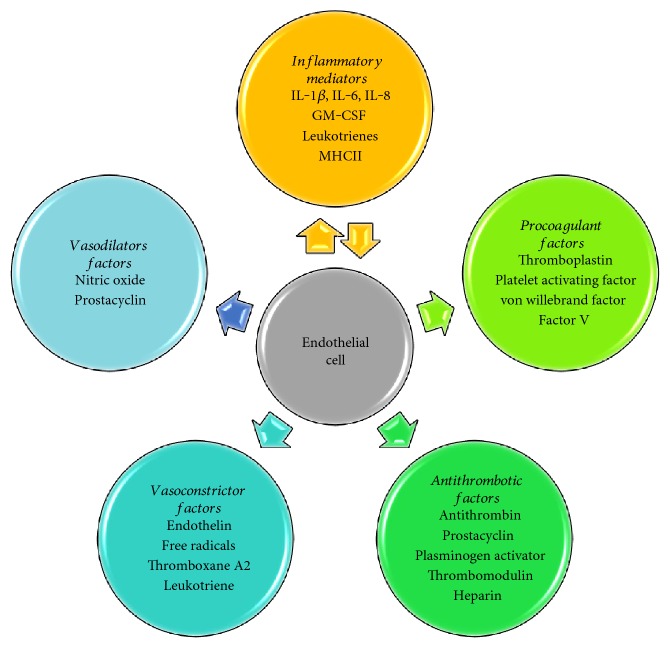
Principal molecules expressed and produced by endothelial cells that are involved in the control of vascular tone, blood flow, hemostasis, and proinflammatory responses.

At the luminal side (inner side), the endothelium is exposed to blood components and serves as a containment barrier for blood components, while on its external face, endothelial cells are directly communicated with smooth muscle cells or pericytes by myoendothelial gap junctions, allowing transfer of ions and small molecules such as calcium to supply metabolic needs [2, 17]. Three types of intercellular junctions between the adjacent endothelial cells have been described: they are tight, gap, and adherent junctions. Their distribution changes along the vasculature because the expression and organization of these connections depends on vessel size and permeability requirements of the perfused organs [17]. Additionally, the endothelium is considered the main regulator of vascular homeostasis that controls vascular tone, blood flow, angiogenesis, and hemostasis and in some cases regulating thrombosis, thrombolysis, and platelet adhesion [1, 2]. All these responses occur in the presence of different stimuli such as hormones, cytokines, and physical and chemical changes (e.g., changes in pressure, pH).  Figure 1 shows the main molecules secreted by the endothelium that are involved in vascular homeostasis.

Changes that affect the endothelium function include the increase of oxidative stress, reduction of NO bioavailability, fluctuations in blood pressure, alterations in prostanoid production, increase of endothelin production, decline of the endothelial cells hyperpolarization, among others [6]. The term “endothelial dysfunction” was established in the early eighties after Furchgott and Zawadzki discovered that the effect of acetylcholine in the relaxation of vascular smooth muscle requires the presence of endothelial cells [18]. Endothelial dysfunction was initially described as an early event that trigger defects in the vascular wall and was strongly associated with the development of atherosclerosis in humans [19]. However, this term is not only related to hypertensive changes but also refers to damage processes of endothelial cells with physiological and pathological aging processes such as kidney damage, intravascular coagulation, diabetes, obesity, atherosclerosis, hypercholesterolemia, sepsis, trauma, infectious, and inflammatory diseases such as RA, SLE, and vasculitis among others [1, 6, 20].

Although the immune system has been considered a potential inducer of endothelial dysfunction, for example, through the presence of specific autoantibodies against the endothelium (autoimmune responses), the precise etiology of endothelial damage initiation in an inflammatory context is still an enigma. Innate immune responses have not been carefully studied to be directly responsible for the original dysfunction; nevertheless, it is accepted as amplifiers and setters of the endothelial injury [20]. Therefore, it is important to recognize and understand the phases of initiation and progression of endothelial dysfunction in pathologies with inflammatory components, which allows elucidation of potential targets that could be modulated pharmacologically for restoring normal structure and functionality of this tissue.

## 3. The Endothelium and Immune Responses

Endothelial cells are involved in anti- and proinflammatory immune responses because they produce different soluble factors and express adhesion molecules that recognize and allow for leukocyte adherence, rolling, and extravasation [2, 3]. Endothelial cells secrete a variety of cytokines such as IL-1*β*, IL-6, IL-8, and granulocyte-macrophage colony-stimulating factor (GM-CSF) in response to hypoxemia, several bacterial products, and cytokines such as TNF-*α*, among other inducers [2, 3]. These mediators have an effect on cells from the innate and adaptive immune system, mediating their recruitment and cell turnover.

One of the main functions attributed to the endothelium in the context of the immune responses is leukocyte transmigration, from the vascular lumen into the tissues; this is a determinant event for the initiation and resolution of different inflammatory processes. Leukocyte migration involves the contribution of a variety of adhesion molecules, which mediate their direct interaction with endothelial cells, such as lectins CD62P and CD62E and the glycoproteins ICAM-1, ICAM-2, VCAM-1, and CD99 [2, 21–23]. Some general aspects of cell adhesion molecules and chemokines involved in leukocyte-endothelial interaction are summarized in Table 1.

Previous studies have shown that the deposit of specific autoantibodies on the endothelium in murine models of autoimmunity and in vitro approaches with HUVEC may contribute to endothelial damage. Antibodies against endothelial cells (antiendothelial cell antibodies (AECA)) can be potentially pathogenic as they are involved in the activation of endothelial cells; promoting the expression of adhesion molecules like E-selectin, VCAM-1, and ICAM-1; increasing the production of inflammatory cytokines and chemokines (IL-1*β*, TNF-*α*, CX3CL1—fractalkine, etc.); facilitating leukocyte recruitment; and promoting apoptosis and necrosis of endothelial cells [8, 20]. Furthermore, AECA may contribute to the inflammatory process in situ activating complement by the classical pathway and increasing cell cytotoxicity [20].

Caterina et al. in 1995 stimulated human saphenuos vein endothelial cells (HSVEC) with different cytokines (IL-1*α*, IL-1*β*, IL-4, and TNF-*α*) and NO, in order to determine whether this radical modulates the endothelial activation induced by these soluble factors. NO inhibited the expression of VCAM-1 and E-selectin and the secretion of IL-6 and IL-8; in addition, it decreased human monocyte adhesion to the endothelial monolayer. This shows that^.^NO not only is exclusively involved in the maintenance of vascular tone but also restricts endothelial activation possibly contributing to the anti-inflammatory and antiatherogenic state that should characterize vascular walls of healthy individuals with low risk factors of cardiovascular diseases [24].

In addition to diapedesis, it has been proposed that an immune response in situ that favors preservation or mediates endothelial injury might exist in endothelial tissue. MIEC from C57/BL56 mice mediated the transmigration of Ly6C^Lo^ monocytes to affected tissues in response to the toll-like receptor- (TLR-) 4 ligands as lipopolysaccharide (LPS), while TLR-7 ligands (resiquimod) induced the intravascular retention of those monocytes promoting endothelial damage [25]. Therefore, it was proposed that immune endothelial alterations depend on the way these cells are activated. Endothelial compromise can also be mediated by antibodies that form IC in circulation, which could bind to complement, and the Fc receptors on monocytes and neutrophils, producing proinflammatory mediators, reactive oxygen species, and deleterious enzymes [20].

Autoantibodies against neutrophils and monocytes can induce a commitment of these cells, but could also promote endothelial alterations with vascular inflammation. van der Woude et al. in 1985 detected autoantibodies of the IgG isotype against the extracellular components of granulocytes and monocytes in 25 of 27 patients with active granulomatosis with polyangiitis (Wegener's granulomatosis) which had vasculitis, and in 4 of 32 samples from patients with inactive disease [26]. In the last years, it has been described that neutrophils play a pivotal role in the pathophysiology of antineutrophil cytoplasmic antibody- (ANCA-) positive vasculitis, such as in granulomatosis with polyangiitis. These cells can be the source of autoantigens, are activated by the ANCA, and are effector cells of the endothelium damage [27, 28]. Therefore, it is not clear if monocytes could have the same role in this and other autoimmune diseases regarding endothelial dysfunction. The evidences presented until now suggest that intravascular phagocytes and their products play an important role in mediating endothelial damage.

## 4. Monocytes and Endothelial Damage

It has been demonstrated that monocytes play a dual role in endothelial patrolling, depending upon their activation stage. They have monitoring functions and removal of AC, MP, and other debris in a steady state to preserve the endothelium integral structure [29, 30]. Contrarily, under proinflammatory stages, these phagocytes contribute to the secretion of mediators of endothelial damage, such as reactive oxygen species, IL-1*β*, and TNF-*α* [31].

According to CD14 and CD16 expression, the monocytes from human peripheral blood are divided into three subpopulations, CD14^++^CD16^−^ classical, CD14^+^CD16^++^ nonclassical, and CD14^++^CD16^+^ intermediate [32]. The gene profile and chemokine receptor expression showed that Ly6C^+^ CCR2^+^ mouse monocytes correspond with the classical and intermediate human monocytes, while Ly6C^−/Lo^CX3CR1^+^ mouse monocytes correspond to nonclassical human monocytes [33].

**Figure 2:**
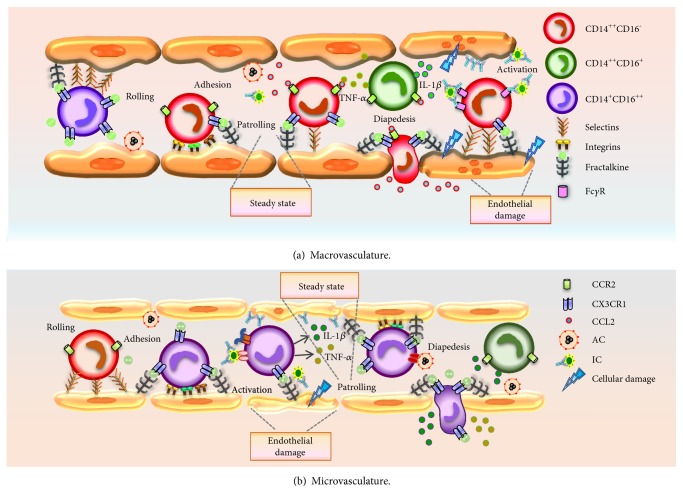
Interaction of monocytes with endothelial cells. Monocyte subpopulations at steady state are involved in maintenance of endothelial integrity by removing MP, AC, and other cellular debris; however, after an inflammatory environment, monocytes may differentially contribute to endothelial damage depending on the subpopulation, kind of stimulus, and endothelium type where immune response is generated. (a) CD14^++^CD16^−^ classical monocytes are preferably adhered to macrovasculature endothelium, patrolling and monitoring large vessels at steady state. Under inflammatory stimuli such as TNF-*α*, which activates endothelial cells, CD14^++^CD16^−^ monocytes migrate to the inflammation site in response to CCL2 (MCP-1) and amplify the inflammatory reaction. CD14^++^CD16^+^ intermediate monocytes are weakly adhered to both kind of endothelium and are mainly producers of IL-1*β* and TNF-*α* after stimulation with TLR4 agonist. (b) CD14^+^CD16^++^ nonclassical monocytes are preferably adhered to microvasculature, patrolling this type of endothelium by interactions with CX3CR1. Depending on the stimulus, for example, in response to bacterial infection or tissue damage, CD14^+^CD16^++^ monocytes can migrate to the inflammation site (by CX3CL1). Please notice that the graph only shows some components of the vascular wall and some membrane proteins that express monocyte subpopulations and endothelial cells.

The recruitment of monocyte subpopulations to different tissues is regulated by endothelial microenvironment involving local production of cytokines and chemokines (Table 1) [34]. Classical and nonclassical monocytes have different migration patterns [34–36]. CD14^++^CD16^−^ monocytes preferentially express CCR2, while CD14^+^CD16^++^ exhibit high expression of CCR5; CCR1 expression is equivalent in both subpopulations [35]. Weber et al. in 2000 demonstrated that sorted CD16^−^ human monocytes migrated through HUVEC endothelial cells in response to MCP-1 (monocyte chemotactic protein-1), and this event was due to the higher expression of CCR2, while the migration of CD16^+^ monocytes in response to MIP-1*α* (macrophage inflammatory protein-1*α*) was due to their higher expression of CCR5 [35] (Figure 2(a)). Ancuta et al. in 2003 found that CD16^+^ monocytes had high levels of CX3CR1 and CXCR4 and low levels of CCR2 and CD62L and exhibited efficient migration through HUVEC cells transfected with a plasmid containing the fractalkine gene (CX3CL1) [36]. These results showed that the CX3CR1/CX3CL1 pathway appears to contribute to the interaction of some monocytes with endothelial cells [36].

Fractalkine (CX3CL1) is the only transmembrane chemokine that functions as a cell adhesion molecule by binding to CX3CR1; however, it may also be cleaved into a soluble fragment which is recognized by the same receptor [23]. Fractalkine expression in the endothelial cells is constitutive and can be induced by inflammatory cytokines, such as TNF-*α*, IL-1*β*, and IFN-*γ* [3]. Fractalkine participates in weak and strong interactions between endothelial cells and leukocytes; with P- and E-selectins, it mediates initial interaction or rolling, while with integrins, it mediates firm adherence and facilitate leukocyte extravasation [23]. It was observed that the CXC3R1 protein and mRNA expression is reduced in total monocytes from sepsis patients, whereas CX3CL1 concentration was elevated in serum, suggesting that monocytes from sepsis patients are retained in circulation because of CX3CL1 levels and CXC3R1 expression, preventing their recruitment to the focus of infection [37].

In the last years, it has been described in the murine model that Ly6C^−/Lo^CX3CR1^+^ monocytes are enriched in the marginal zone of blood vessels. Auffray et al. in 2007 studied the behavior and function of monocytes in vivo in the steady state and under inflammatory conditions; for this propose, they monitored the vasculature of Rag_2_^−/−^ Cx_3_cr1^gfp+^ mice by intravital microscopy, after the adoptive transfer of human monocyte subpopulations labeled with fluorescent probes. At steady state, the human and murine monocytes were in close contact with the endothelium (at the dermis blood vessels and in the branches of the mesenteric artery) and in the presence of the constant blood flow; these cells had a patrolling movement on the blood vessels depending on the LFA-1 and CX3CR1. Extravasation of these cells was not observed during the period evaluated. After proinflammatory stimuli, such as tissue mechanical damage or peritoneal infection with the intracellular bacterium *Listeria monocytogenes*, these monocytes were quickly recruited (one hour) to the affected site, resulting in high levels of TNF-*α* and IL-1*β*. Therefore, researchers proposed that Ly6C^−/Lo^ monocytes may be the first line of cells to respond to the inflammatory events that produces high amounts of proinflammatory cytokines, which could increase the extravasation of further components of the immune system [29].

In another study of the same group, it was found that steady Ly6C^−/Lo^ monocytes patrol the endothelium to remove MP and AC through interactions depending on LFA-1 and ICAM-1 adhesion molecules [25]. Intravascular retention (without diapedesis) of these monocytes was observed in response to pathogen-associated molecular patterns (PAMP) such as a TLR-7 ligand but not with a TLR-4 ligand, in a way that depends on fractalkine expression. In addition, neutrophil recruitment was also promoted to the affected site, with a consequent induction of endothelial damage inside the vascular lumen [25].

Concordantly, there is evidence that suggests that human CD14^+^CD16^++^ (the counterpart of the murine Ly6C^−/Lo^ monocytes) also have close contact with the endothelium [33]. In humans, it was shown that over 75% of CD14^+^CD16^++^CX3CR1^+^ monocytes are found in the marginal zone of the blood vessels. These cells apparently reside in this location and interact with endothelial cells by the expression of a variety of adhesion molecules such as CD11b and VLA-4. These monocytes are mobilized from the marginal zone of the vessels by nonspecific stimuli such as anaerobic exercise, increasing their circulating numbers in minutes [38].

Cros et al. in 2010 characterized the functions of human CD14^+^CD16^++^ in samples isolated from healthy people and MYD88 or IRAK-4-deficient patients (autosomal recessive defect); they compared these results with murine Ly6C^−/Lo^ monocytes. At steady state, the human monocytes showed an anti-inflammatory profile; but after infection with different viruses (herpes simplex type I and measles) and treatment with IC containing nucleic acids, these cells produced TNF-*α*, IL-1*β*, and CCL3, through the stimulation of TLR7, TLR8, MyD88, and MEK activation pathways. Until now, results have suggested that the CD14^+^CD16^++^ monocytes patrol the endothelium, detecting viral infections and IC and producing proinflammatory cytokines in response to these stimuli. Furthermore, in this research, it was proposed that the activation of monocytes by IC and nucleic acids could contribute to autoimmune disease pathogenesis such as SLE, because of the accumulation of IC in the microvasculature of different tissues, particularly in the renal glomerulus [33] (Figure 2(b)).

Collison et al. in 2015 evaluated the locomotion of monocyte subpopulations during adhesion to endothelial cells using human cells. After the separation of three monocyte subpopulations from healthy persons, the cells were cocultured with macrovasculature (HUVEC) and microvasculature (human dermal blood endothelial cells (HDBEC)) endothelial cells under a shear flow system. Each subpopulation showed different locomotion patterns depending on the type of endothelial cell. CD14^++^CD16^−^ monocytes were preferably adhered to HUVEC, arrested in the monolayer, and showed patrolling behavior, while CD14^++^CD16^+^ and CD14^+^CD16^++^ monocytes did preferably bond to HDBEC. Only the CD14^+^CD16^++^ monocytes crawled long distances exerting a patrolling movement. Each form of locomotion had a different requirement for adhesion molecules; in particular, the long-range crawling behavior in CD14^+^CD16^++^ monocytes was abrogated by blockade of ICAM1, VCAM1, or CX3CL1, whereas this behavior in CD14^++^CD16^−^ monocytes was stopped by blockade of ICAM1. Upon activation of the endothelial cells by TNF-*α*, the expression of CX3CL1 increased in the macro- and microvasculature; hence, the migration of CD14^++^CD16^−^ and CD14^+^CD16^++^ monocytes was observed. This study demonstrated the differential behavior and locomotion heterogeneity that monocyte subpopulations present in endothelial cells before and after activation stimuli [30] (Figure 2).

These evidences show that both murine and human monocytes interact directly with the endothelium, they have surveillance and patrolling functions, and these functions apparently depend on the subpopulation involved. However, under certain stimuli and proinflammatory responses, these phagocytes could participate in endothelial damage. Little is known of how these monocytes interact with micro- and macrovasculature endothelial cells in the context of RA and SLE and if they could be contributing to endothelial dysfunction in these pathologies.

## 5. Endothelial Alterations in SLE and RA: Potential Contribution of Monocytes

Endothelial alterations in both micro- and macrovasculature have been demonstrated in patients with RA and SLE evaluated by FMD (flow motion dilation) of the brachial artery, assessing arterial stiffness and thickening of carotid intima-media (the intima-media thickness (IMT)), among other tests [39]. These studies have shown that these vascular changes are positively correlated with the inflammatory status of patients using the measurement of acute phase proteins (C-reactive protein and IL-6), with chronic use of corticosteroids and clinical activity of the diseases [40]. In fact, RA and SLE patients have an endothelial dysfunction associated with an increased risk of developing atherosclerosis and cardiovascular diseases, which reduce their life expectancy by 10–15 years [5]. In general, the pathways and mechanisms involved in the initial endothelial damage in SLE and RA patients are still not known; however, it is postulated that it can be partly due to the immune system activation, chronic inflammation, and oxidative stress presented in these individuals. The immunological mechanisms which can lead to endothelial dysfunction in SLE and RA included mainly type II (cytotoxic reactions) and type III (immune complex injury) hypersensitivity reactions; however, it is possible that other mechanisms can also activate the immune system and promote inflammation in these autoimmune diseases [41, 42].

### 5.1. Endothelial Alterations in SLE

SLE is a chronic systemic autoimmune disease, characterized by loss of immunological tolerance of B cells, with the subsequent autoantibody production against double-stranded DNA, nuclear proteins such as Smith (Sm) and phospholipids, among others [8, 9]. These antibodies may directly bind to endothelial cells or are part of the circulating IC that can be deposited in the vessels, promoting inflammatory responses of innate immune cells and increasing endothelial permeability and leukocyte infiltration to the affected tissues [9]. In addition, the continuous exposure of patrolling monocytes of these patients to circulating autoantibodies and IC may also lead to chronic endothelial cell activation mediated by the interaction with monocytes, causing injuries of blood vessels and vasculitis [43].

Pentraxin 3 (PTX3), which is produced by endothelial cells in response to various inflammatory events, has been proposed as an indicator of inflammatory vascular injury and as a biomarker of vasculitis in SLE and other diseases such as sepsis, septic shock, and preeclampsia. This protein binds with high affinity to C1q and it is involved in repairing the blood vessels, mediating angiogenesis, atherosclerosis, and restenosis, among others [43]. The PTX3 levels and other indicators of endothelial dysfunction (like the soluble form of E-selectin (sE-selectin), VCAM-1 (sVCAM-1), MCP-1 (*monocyte chemotactic protein-1*), and von Willebrand factor (vWF)) were estimated in plasma and serum of 56 women with SLE. These patients had high concentrations of PTX3, vWF, MCP-1, sE-selectin, and sVCAM-1 compared to healthy controls. The expression levels of PTX3 were also associated with the activity index, the prednisolone dose received, the severity of anemia present in those patients, and vWF and sVCAM-1 levels. Therefore, it was concluded that the concentration of PTX3 may be an indicator of endothelial activation or dysfunction in SLE patients [43].

Daha et al. in 1988 showed that the purified human C1q labeled with iodine 125 (I-125) could interact with HUVEC by collagen binding region and maybe through PTX3. When HUVEC was incubated with IC formed by bovine thyroglobulin (BTg) and rabbit antibodies against BTg, the binding of those IC to endothelium increased with the presence of C1q [44]. This suggests that antibodies forming IC with specificities other than endothelium molecules could also deposit on endothelial cells through a classical complement component, boosting innate immune response.

Other studies reported that alterations in the coagulation and thrombus formation in SLE patients is associated with the presence of antiphospholipid antibodies: anticardiolipin, anti-*β*2-glycoprotein and LA [9]. These antibodies bind to the negative surface of the phospholipids in endothelial cells, preventing the union of the inhibitors of coagulation, like the tissue factor pathway inhibitor (TFPI) or the protein C-protein S complex to the cell surface, hence causing the activation and aggregation of platelets and thrombus formation. These antibodies could also induce endothelial damage in a similar way. In fact, it was demonstrated that some specific autoantibodies against phospholipids can activate endothelial cells, inducing membrane expression of ICAM-1, VCAM-1, and E-selectin and also increases the expression of tissue factor in endothelial cells and monocytes from SLE patients [45].

**Figure 3:**
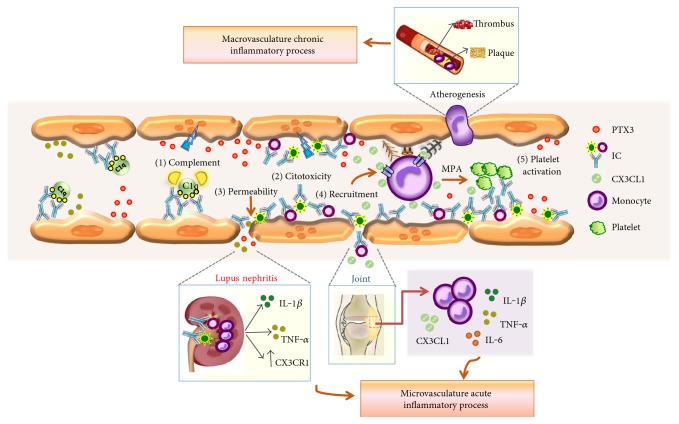
Endothelial alterations in SLE and RA. The binding of autoantibodies to the endothelium and deposition of IC on the microvasculature lead to classical complement and monocyte activation and increased endothelial permeability by alterations of interendothelial junctions. There is also increased cell cytotoxicity by immune cells, which further affects the integrity of the tissue. This endothelial activation and damage produce an acute inflammatory response, which recruit further innate immune cells as neutrophils and other monocytes, induce platelet aggregation with the consequent procoagulant activity and microthrombus formation. These inflammatory events can affect different organs and tissues such as kidney in LES (red) and joint in AR (blue), which contribute to the pathogenesis of these diseases. Finally, the persistence of these inflammatory events could conduce to a macrovascular endothelial alterations and chronic inflammatory process that leads to the development of complications in larger vessels, such as atherosclerosis and cardiovascular disease. Please notice that the graph only shows some components of the vascular wall and some membrane proteins that express monocyte subpopulations and endothelial cells; in addition, it is important to clarify that the graph does not show differences between macro- and microvasculature.

Martini et al. in 1996 investigated the presence of phospholipids antibodies, anticardiolipin (aCL), and lupus anticoagulant (LA) in serum of 22 SLE patients by ELISA and KCT (*kaolin clotting time*) coagulation test. The results showed that 54.5% of SLE patients were positive for LA, 64% for aCL, and 59% for both factors (aCL and AL). Subsequently, the tissue factor productions by monocyte (monocyte procoagulant activity (MPA)) from the controls exposed to plasma from SLE patients were evaluated. The MPA was significantly increased with the serum from patients who were aCL-positive and/or LA+, compared with SLE patients without these autoantibodies. These results show that the presence of aCL and/or LA in SLE patients is associated with the increase in monocyte activation, thereby promoting the occurrence of thrombotic events in these patients [46]. The results also suggest that this class of autoantibodies could mediate endothelial dysfunction indirectly by inducing activation of monocytes (Figure 3).

AECA have been found in the sera from SLE patients; however, the mechanism by which these antibodies are involved in the development of endothelial dysfunction is not yet completely understood. Moscato et al. in 2002 separated anti-DNA and other autoantibodies from the serum of SLE patients by affinity chromatography and assessed their binding to HUVEC cells using immunoprecipitation and flow cytometry. Both classes of antibodies recognized different membrane components of the endothelial cells; however, this binding did not induce cytotoxicity by complement or apoptosis, suggesting that anti-DNA and other autoantibodies can recognize the surface of HUVEC but do not appear to be directly responsible for endothelial dysfunction [8]. However, if these antibodies are bound to the endothelium and are recognized by the patrolling monocytes, it would promote the damage of these cells.

Renal involvement includes the most serious affections of SLE. Monocytes, macrophages, and T lymphocytes play a critical role in the initiation and progression of lupus nephritis; these cells can cause an increase in endothelial permeability by proinflammatory cytokine production and cytotoxic reactions (by autoantibodies or complement system) that allow the immune cell infiltration of the glomeruli and interstitium and finally kidney damage [47]. Yoshimoto et al. in 2007 studied the association among the fractalkine expression in the glomerular capillaries, the infiltration of CD16^+^ monocytes in response to this chemokine, and the severity of glomerular lesions in patients with renal involvement. They collected renal biopsies from patients with different kinds of nephritis (I–V), and performed histopathological and immunohistochemical studies, as well as RNA extraction and RT-PCR (*reverse transcription polymerase chain*). Patients with proliferative lupus nephritis (class III and IV) had significantly higher expression of fractalkine and higher amount of CD16^+^CX3CR1^+^ monocyte infiltration than the control biopsies. Furthermore, the glomerular fractalkine expression correlated significantly with histopathologic activity index and the amount of CD16^+^ monocytes; this last variable also correlated with serum levels of urea, complement, and anti-DNA. These findings suggest that monocytes appear to respond effectively to changes observed in the endothelium, hence contributing to disease pathology; however, the initial stimuli that triggers the endothelial activation, dysfunction, and nephritis in SLE is still unknown [47].

Mikolajczyk et al. in 2015 investigated the relationship between atherosclerosis, endothelial dysfunction, and monocyte phenotype in SLE patients [48]. They characterized the monocyte subpopulations in peripheral blood samples from 42 SLE patients and determined the IMT of the carotid arteries (with ultrasonographic) as an indication of atherogenesis, and FMD and NMD (nitroglycerin-induced dilation) as indicators of endothelial dysfunction. SLE patients showed increased thickness of carotid arteries and endothelial dysfunction when compared to controls by IMT and FMD, respectively; IMT data correlated positively with an increase in the frequency of CD14^+^CD16^++^ subpopulation. In addition, an increase in CD14^++^CD16^+^ monocytes was observed in SLE patients compared to healthy controls [48]. Thus, it could be proposed that CD14^+^CD16^++^ monocytes are related to atherosclerosis in SLE patients; however, it is not clear whether these cells could be involved in the endothelial dysfunction that occurs in this disease.

The evidences have shown that endothelial alterations in SLE can be associated with autoantibodies against phospholipids that activate endothelial cells and cause damage (type II hypersensitivity reaction), also circulating IC that induce innate immune responses, including monocytes, and trigger type III hypersensitivity reactions. Although it is clear that monocyte subpopulations in SLE have different alterations in their proportion in circulation, little is known about the changes in their phenotype, the interaction they have with endothelial cells, and their role in the development of endothelial dysfunction and atherosclerosis in this disease. Therefore, it is proposed that the activation of patrolling monocytes, for example, by IC recognition, could induce classical activation of these cells with consequent induction of a proinflammatory environment. This would begin endothelial dysfunction in macrovasculature and atherosclerosis, as well as contribute to the damage of endothelium from microvasculature in different target organs such as the skin and kidney (Figure 3).

### 5.2. Endothelial Alterations in RA

RA is a chronic systemic inflammatory disease characterized by persistent involvement of different joints. RA patients have a high risk of developing atherosclerosis and consequently cardiovascular disease; 30–50% of RA patients die for these causes. This is partly because these patients have different endothelial alterations, such as persistent activation, prothrombotic properties, and a reduction on vasodilator response [49–51]. Bergholm et al. in 2002 were the first to identify endothelial dysfunction in RA patients. They found that the vasodilator response of the intrabrachial artery to acetylcholine and sodium nitroprusside infusions was lower in 10 newly diagnosed RA patients (maximum 18 months after the diagnostic) than the response of healthy controls. This suggests the presence of early endothelial dysfunction in these patients [50].

The endothelial dysfunction has been described as an integral part of the pathogenesis of RA, because the inflammatory condition that occurs in this disease primarily affects small and medium vessels (microvasculature), causing even rheumatoid vasculitis (RV). These patients have a vast array of clinical manifestations with a predilection for the skin (peripheral gangrene, deep cutaneous ulcers) and the peripheral nervous system (mononeuritis multiplex). Systemic vasculitis in RA has been associated with high levels of rheumatoid factor (RF) and increased amounts of anti-C1q, AECA, and anti-glucose-6-phosphate isomerase, among others [52], suggesting that similar to SLE, the autoantibodies apparently perform an important role in the endothelial alterations. Siegert et al. in 1990 investigated the presence of IgG and IgA antibodies that bound to C1q (as a measure of circulating IC) in serum samples from 80 RA patients without RV, 31 patients with RV, and 80 healthy controls. The IgG and IgA antibodies bound to C1q were detected in only 5% of RA patients without RV, while 29% and 61% of patients with RV had IgG and IgA antibodies bound to C1q, respectively. Therefore, it was shown that IgG and IgA antibodies contribute to the formation of circulating IC in RA patients and suggest that they are associated with the development of RV [53].

Increased levels of endothelial progenitor cells (EPC) were observed in the synovial fluid of RA patients compared with circulation levels of the same patients. This recruitment of the EPC to synovia was associated with blood vessel formation in this region (angiogenesis) and with a reduction in the number of these cells in circulation. This could increase cardiovascular risk, because it possibly disturbs the ability of these patients to repair the endothelium and to do revascularization of the ischemic and affected areas [40].

It is estimated that 60% of RA patients suffer chronic synovitis that eventually destroys the joint. It has been suggested that circulating monocytes play an important role in chronic synovitis, because they primarily infiltrate the joint and produce inflammatory cytokines (IL-1*β*, IL-6, and TNF-*α*) [31]. Grober et al. in 1992 evaluated mononuclear cell interaction with microvasculature endothelial cells, which were obtained from knee synovial tissue or the hip of patients with chronic RA after arthroplasty. The endothelial cells were incubated with monocytes or lymphocytes obtained from peripheral blood of the patients, and the cell-cell interaction were analyzed by confocal microscopy (binding assay Stamper-Woodruff). The observation revealed that the monocytes were bound more frequently to the endothelium than the lymphocytes; in addition, blocking P-selectin with a neutralizing antibody reduced more than 90% of the monocyte adhesion to the synovial microvasculature, while no change was observed by blocking E-selectin, L-selectin, LFA-1, and *β*2 integrin. This study suggests that the interaction of monocytes with endothelial cells of RA patients by P-selectin could mediate the infiltration of these phagocytes to synovial tissue [31]. However, it is important to demonstrate if those interactions could promote the endothelial alterations of these patients.

Ruth et al. in 2001 evaluated the expression of CX3CL1 and CX3CR1 receptor by immunohistochemistry in experimental arthritis model associated with *Mycobacterium butyricum* in Lewis rats. The macrophages, fibroblasts, and dendritic cells at the synovial tissue expressed both fractalkine and its receptor, whereas the endothelial cells only expressed CX3CL1. Furthermore, an increase in CX3CR1 and CX3CL1 mRNA was also found in the ankle when rats showed severe inflammation [54]. The same authors also evaluated the expression of CX3CL1 and its receptor in peripheral blood samples and synovial fluid of patients with RA, osteoarthritis, juvenile rheumatoid arthritis, psoriatic arthritis, polyarthritis, spondyloarthropathy, inflammatory myopathy, and gout. They found similar results, showing that macrophages, fibroblasts, endothelial cells, and dendritic cells expressed CX3CL1; in addition, the macrophages and dendritic cells were also positive for CX3CR1. By ELISA, they observed high levels of soluble fractalkine fragment at the synovial fluid of RA patients, compared to patients with osteoarthritis and other forms of arthritis. The in vitro blockade of fractalkine presented in synovial fluid of RA patients by anti-human specific antibody significantly decreased its capability to induce monocyte migration under different chemotactic assays. These results clearly showed the relevance of this chemokine and its receptor in the monocyte migration through endothelium in the context of RA [54]. However, it is not yet known whether this pathway could be involved in the endothelial dysfunction of these patients.

ADAM (a disintegrin and metalloproteases) is a family of proteases that release a variety of membrane proteins; it has been reported that ADAM-10 is responsible for releasing different chemokines, like CXCL16 and CX3CL1 [55]. Isozaki et al. in 2015 reported that the levels of ADAM-10 in serum from RA patients were significantly higher compared to healthy controls by ELISA; this correlated with the disease activity measured by DAS28 (disease activity score of 28). The treatment of the synovial fluid from RA patients with a specific antibody against ADAM-10 decreased in vitro migration of THP-1 cells and monocytes from healthy individuals. These findings were corroborated with the fibroblast-like synoviocytes from patients, which were transfected with specific siRNA (small interfering RNA) to ADAM-10 transcription; the silencing of this gene in vitro inhibited the monocyte and synoviocyte adhesion to the endothelial cells and decreased growth factor and production of CX3CL1. These results suggest that ADAM-10 plays an important role in the monocyte adhesion to inflamed tissues from RA patients and that it could be involved in the endothelial alteration of these patients [55].

In summary, endothelial alterations can occur in RA patients both in early and chronic states; these can arise in the microvasculature by IC deposition, causing articular involvement, deterioration of synovial joints, and in some cases, a persistent vasculitis (type III hypersensitivity reactions). However, type II mechanisms had also been involved in the endothelial damage of this disease. Endothelial dysfunction also appears to affect larger vessels of these patients leading to increased atherosclerosis and the development of a chronic inflammatory process. It is possible that the activation of patrolling monocytes, for example by IC, participate in these endothelial abnormalities and increases the vascular permeability; this could facilitate the articular inflammatory response and the damage of endothelial cells from macrovasculature that conduce to plaque formation in RA (Figure 3). Although it has been described that different molecules can participate in the monocyte-endothelial interaction in the context of RA, the exact molecular mechanism involved and whether this interaction can promote endothelial damage is not yet known. Finally, the apparent inability of these patients to repair the endothelium could further perpetuate this tissue damage and increase the risk of suffering vascular complications.

## 6. Conclusions and Perspectives

RA and SLE are autoimmune diseases that clearly have micro- and macrovasculature endothelial alterations. Different organs and tissues such as kidney, joints, skin, among others are severely affected by the inflammation of the endothelium. The endothelial damage that occurs in these patients is related to the persistent inflammatory response that characterizes these diseases and is associated with the presence of autoantibodies, immune complexes, and monocytes activation. It is considered that endothelial compromise in RA and SLE is due to the direct binding of autoantibodies to the endothelial cells which in turn promote type II hypersensitivity reactions, as well as the deposit on the endothelium of IC in a soluble form or as part of AC and MP (type III hypersensitivity reactions). Both mechanisms can increase endothelial permeability and complement-dependent cytotoxicity and decrease endothelial junctions, leading to a secondary apoptosis of these cells. Finally, these responses induce prothrombotic activity and leukocyte recruitment to different tissues, resulting in loss of integrity and function of the endothelium and underlying tissues (Figure 3). It is considered that some of these responses could also contribute to the initial endothelial damage that are required for the accumulation of low density lipoprotein and formation of atherosclerotic plaques in the arterial intima of medium and larger vessels, developing a chronic macrovascular inflammatory process.

We proposed that monocytes subpopulations must be differentially involved in the initial damage mechanisms of micro- and macrovasculature. Therefore, depending of the endothelium, a monocyte subpopulation could differentially participate in the inflammatory process mediating endothelial damage. Finally, high concentrations of molecules like PTX3 and CX3CL1 could be used as a biomarker of activation and endothelial dysfunction in SLE and RA patients. The mechanisms described here are possibly involved in endothelial alterations of these diseases; however, further studies are required in order to better understand the role of monocytes and their molecules in the endothelial dysfunction of RA and SLE patients.

Given the role of the endothelium in the pathophysiology and complications of SLE and RA, as well as other inflammatory entities in human, it will be important to get a better characterization and understanding of the role played by monocyte subpopulations and IC in the induction of endothelial damage. This could be useful to get new approaches and therapeutic interventions that can modulate monocyte responses and interaction with endothelial cells and also to reduce or prevent endothelial alterations in these diseases or other chronic inflammatory diseases where there is also a damage of this tissue (Figure 2).

## Figures and Tables

**Table 1:** Molecules involved in the interaction between leukocytes and endothelium [35, 36].

Family	Molecule	Cellular distribution	Ligand cell type
Selectin	P-selectin (CD62P)	Endothelium activated by histamine or thrombin	Sialyl Lewis X in PSGL-1 (*P-selectin glycoprotein * *ligand-1*) present in neutrophils, monocytes, and T cells (effector and memory) [56].
	E-selectin (CD62E)	Endothelium activated by cytokines (TNF-*α*, IL-1*β*)	Sialyl Lewis X in CLA-1 (*cutaneous* *lymphocyte-associated antigen-1*) present in neutrophils, monocytes, and T cells (effector and memory) [23].
	L-selectin (CD62L)	Neutrophils, monocytes, and T and B cells in constitutive form	Sialyl Lewis X/PNAd in GlyCAM-1 (*glycosylation-* *dependent cell adhesion molecule-1*), CD34, and MadCAM-1 (*mucosal vascular address in cell * *adhesion molecule 1*) present in endothelium [57].

Immunoglobulin	ICAM-1 (CD54)	Endothelium activated by cytokines (TNF-*α*, IL-1*β*), macrophages, and lymphocytes	LFA-1 (CD11a/CD18) in neutrophils, monocytes, T cells (naive, effector and memory), and B cells (naive).
			Mac-1 (CD11b/CD18) in neutrophils, monocytes, and dendritic cells [2].
	ICAM-2 (CD102)	Endothelium in a constitutive form	Similar to ICAM-1 [2].
	VCAM-1	Endothelium activated by cytokines (TNF-*α*, IL-1*β*)	VLA-4 (CD49a/CD29) in neutrophils, monocytes and T cells (naive, effector and memory).
			*α*_4_ *β*_7_ (CD49d/CD29) in monocytes, T cells (naive, effector and memory), and B cells [58].

Chemokine	CX_3_CR1	T cells, monocytes, and NK cells in a constitutive form	CX_3_CL1 in endothelial cells [36].
